# A Rare Case of Candida-Induced Acute Phlegmonous Esophagitis Complicated by Pericarditis and Myocarditis

**DOI:** 10.7759/cureus.84447

**Published:** 2025-05-20

**Authors:** Kenta Date, Taichi Kato, Yuhei Takuma, Kazuhiro Sugiyama

**Affiliations:** 1 Tertiary Emergency Medical Center, Tokyo Metropolitan Bokutoh Hospital, Tokyo, JPN

**Keywords:** candida esophagitis, immunocompromised host, pericarditis, phlegmonous esophagitis, sjögren's syndrome

## Abstract

Acute phlegmonous esophagitis is a rare, life-threatening condition characterized by inflammation of the submucosal and muscular layers of the esophagus. While bacterial infections are the most common etiology, fungal causes are exceedingly rare and poorly documented.​ ​​We report the case of a 50-year-old immunosuppressed man with multiple autoimmune diseases, including Sjögren’s syndrome, primary biliary cholangitis, and autoimmune hepatitis. He had been receiving long-term corticosteroid therapy for disease control. He developed progressive dysphagia followed by acute chest pain and septic shock. Contrast-enhanced computed tomography revealed diffuse esophageal wall thickening with periesophageal fluid accumulation, and endoscopy showed mucosal erythema and circumferential white plaques in the esophagus. Histopathological examination of biopsy specimens confirmed *Candida* species as the causative organism. Despite the development of pericarditis and myocarditis, early administration of broad-spectrum antibiotics and antifungal therapy led to clinical improvement. This case represents the first documented instance of *Candida*-induced phlegmonous esophagitis and highlights the importance of considering fungal infections in the differential diagnosis, particularly in immunocompromised patients. Early endoscopic evaluation, histological confirmation, and continuous cardiac monitoring are crucial for accurate diagnosis, management, and detection of cardiac complications.​​​

## Introduction

Acute phlegmonous esophagitis is a rare and life-threatening condition characterized by inflammation of the submucosal and muscular layers of the esophagus [1,2]. While phlegmonous gastritis is more commonly reported, phlegmonous involvement of the esophagus remains poorly understood. Established risk factors include immunosuppression, diabetes, alcohol use, and malnutrition [3,4]. Bacterial infections are the usual etiologies, and fungal involvement is extremely rare [1]. Although superficial *Candida* esophagitis is a well-recognized entity in immunocompromised individuals [5], its potential to invade the deeper layers of the esophagus and cause phlegmonous esophagitis has not been clarified. This represents a significant gap in current medical understanding [6]. Based on a systematic search of PubMed using relevant keywords, no previous cases of *Candida*-induced phlegmonous esophagitis were identified. This suggests that under conditions of prolonged immunosuppression, such as in patients with autoimmune diseases like Sjögren’s syndrome, *Candida* may act as an invasive pathogen and contribute to severe esophageal infection. This case report presents what appears to be the first documented instance of Candida-induced phlegmonous esophagitis, complicated by septic shock, pericarditis, and myocarditis and emphasizes the importance of considering fungal pathogens in the differential diagnosis of esophageal infections, alongside the necessity for early endoscopic evaluation and cardiac monitoring.

## Case presentation

The current report discusses our findings in a 50-year-old male patient, diagnosed one year prior with Sjögren’s syndrome and an overlap syndrome of primary biliary cholangitis (PBC) and autoimmune hepatitis, who had been receiving oral prednisolone therapy at 12.5 mg/day for autoimmune control. His medical history included Hashimoto thyroiditis and emphysema. Current medications included prednisolone 12.5 mg (2.5 tablets of 5 mg each), sulfamethoxazole-trimethoprim (400/80 mg), levothyroxine 112.5 μg (4.5 tablets of 25 μg each), ursodeoxycholic acid (900 mg), lansoprazole (15 mg), aspirin (81 mg), isoleucine-leucine-valine (4.15 g), potassium gluconate (10 mEq), L-carbocisteine (1000 mg), indacaterol-glycopyrronium (inhalation), zinc acetate (150 mg), and vilanterol-fluticasone (inhalation). Sjögren’s syndrome is associated with chronic lymphocytic infiltration and salivary gland dysfunction, which may compromise mucosal barriers and increase susceptibility to infections, particularly in the upper digestive tract.

**Figure 1:**
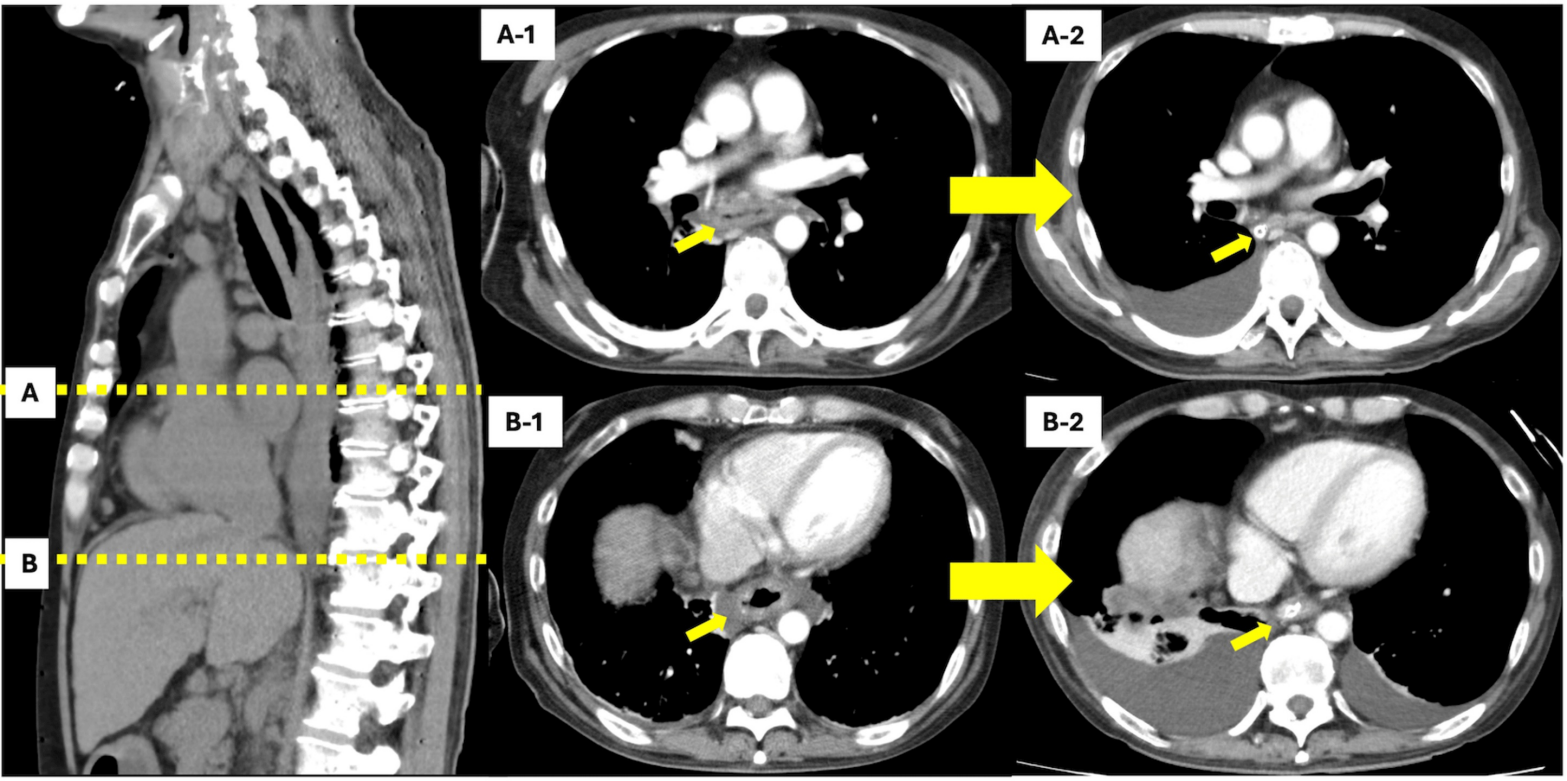
Temporal changes in computed tomography (CT) findings of acute phlegmonous esophagitis A-1 and B-1 show CT images obtained on day 1, revealing esophageal wall thickening and periesophageal inflammation. A-2 and B-2 show CT images on day 9, demonstrating improvement in inflammatory findings.

The patient had experienced progressive dysphagia over three months, initially for solid foods and subsequently for liquids, but did not seek medical attention. On the day of admission, he had worked until 08:00 despite mild left anterior chest pain. After returning home, he slept and awoke at 18:00 with a worsening chest ache, prompting transportation to our emergency department. On arrival, his vital signs were as follows: Glasgow Coma Scale E4V5M6, heart rate 126 bpm, blood pressure 72/40 mmHg, respiratory rate 17 breaths/min, oxygen saturation 92% on room air, and body temperature 37.1°C. Electrocardiography (ECG) and echocardiography showed no acute ischemic changes or pericardial effusion. Arterial blood gas analysis revealed metabolic acidosis (pH 7.302, bicarbonate 17.8 mEq/L) and elevated lactate (3.3 mmol/L), indicating early septic shock characterized by hypotension and elevated lactate levels. Laboratory results are summarized in Table 1, highlighting leukopenia, anemia, hypoalbuminemia, and elevated inflammatory markers. Contrast-enhanced computed tomography (CT) showed diffuse esophageal wall thickening with periesophageal fluid collection (Figure 1).

**Table 1:** Laboratory test results at the time of admission of the patient

Laboratory test	Result	Reference value
White blood cells (x10^3^/μL)	1.6	3.3–8.6
Red blood cells (x10^6^/μL)	2.69	3.86–4.92
Hemoglobin (g/dL)	9.8	11.6–14.8
Platelets (x10^3^/μL)	266	158–348
Fasting blood glucose (mg/dL)	93	73–109
Albumin (g/dL)	1.7	4.1-5.1
Urea (mg/dL)	32.8	8.0–20.0
Serum creatinine (mg/dL)	0.91	0.46–0.79
Serum sodium (mEq/L)	135	138–145
Serum potassium (mEq/L)	3.9	3.6–4.8
Total bilirubin (mg/dL)	4.95	0.40-1.50
Aspartate aminotransferase (AST) (U/L)	50	13-30
Alanine aminotransferase (ALT) (U/L)	53	10-42
C-reactive protein (CRP) (mg/dL)	18.38	0.00-0.14

**Figure 2:**
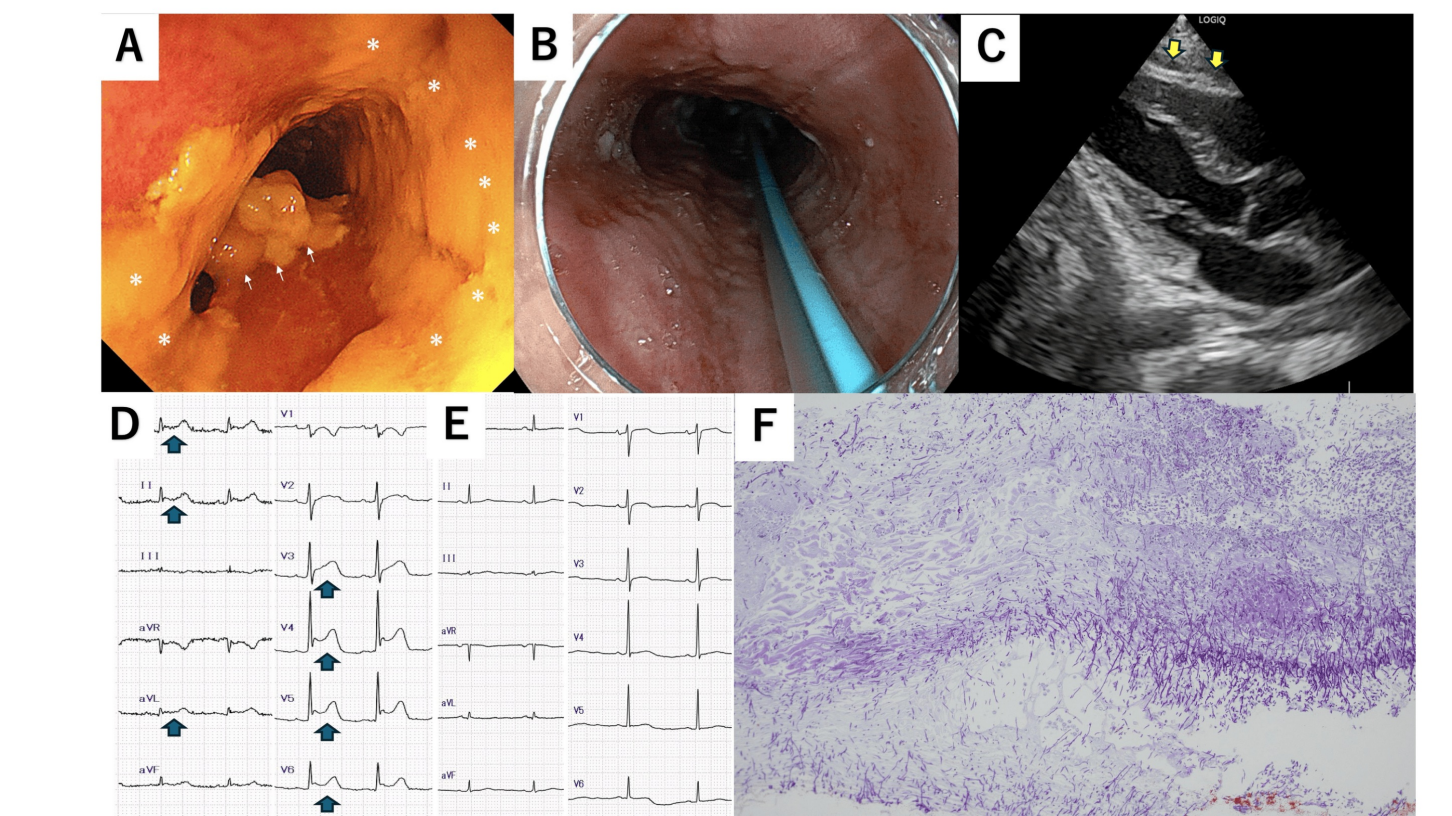
Endoscopic, cardiac, and histopathological findings in a case of acute phlegmonous esophagitis (a) Upper gastrointestinal endoscopy findings on day 1. The asterisk and arrow indicate circumferential white plaques attached to the esophageal wall. (b) Upper gastrointestinal endoscopy findings on day 9. The blue tube is a nasogastric tube. (c) Echocardiography findings on day 2. No pericardial effusion is observed. (d) Electrocardiogram (ECG) on day 2. ST elevations are noted in leads I, II, aVL, and V3–V6. (e) ECG on day 5. ST elevations are absent. (f) Histopathological specimen obtained via upper gastrointestinal endoscopy on day 1. Numerous filamentous fungi are observed, suggesting *Candida *involvement.

Following CT, the patient’s blood pressure further declined, necessitating norepinephrine (0.2 μg/kg/min) and vasopressin (2 U/h) infusions. The patient was intubated and placed on mechanical ventilation under sedation. Upper gastrointestinal endoscopy revealed mucosal erythema and edema from the pharynx to the stomach and circumferential white plaques in the esophagus (Figure 2A). Biopsies were obtained from affected areas. Based on clinical findings, the patient was diagnosed with septic shock secondary to acute phlegmonous esophagitis. Treatment included *nil per os*, fluid management, and empiric therapy with meropenem (MEPM), vancomycin (VCM), and micafungin (MCFG), following blood culture collection.

On day 2 of hospitalization, laboratory tests demonstrated an elevated troponin I (Trop I) level, prompting repeat ECG and echocardiography investigations. The ECG revealed ST elevation in leads I, II, aVL, and V3-V6 (Figure 2D). Although pericarditis was suspected, echocardiography showed no pericardial effusion; thus, pericardiocentesis was not performed (Figure 2C). Antibiotic therapy was continued, and daily follow-up with ECG and echocardiography was conducted. On day 3 of hospitalization, the patient received total parenteral nutrition (TPN) via a central venous catheter, and small amounts of glucose-fructose-oligosaccharides were administered via a nasogastric tube. 

**Figure 3:**
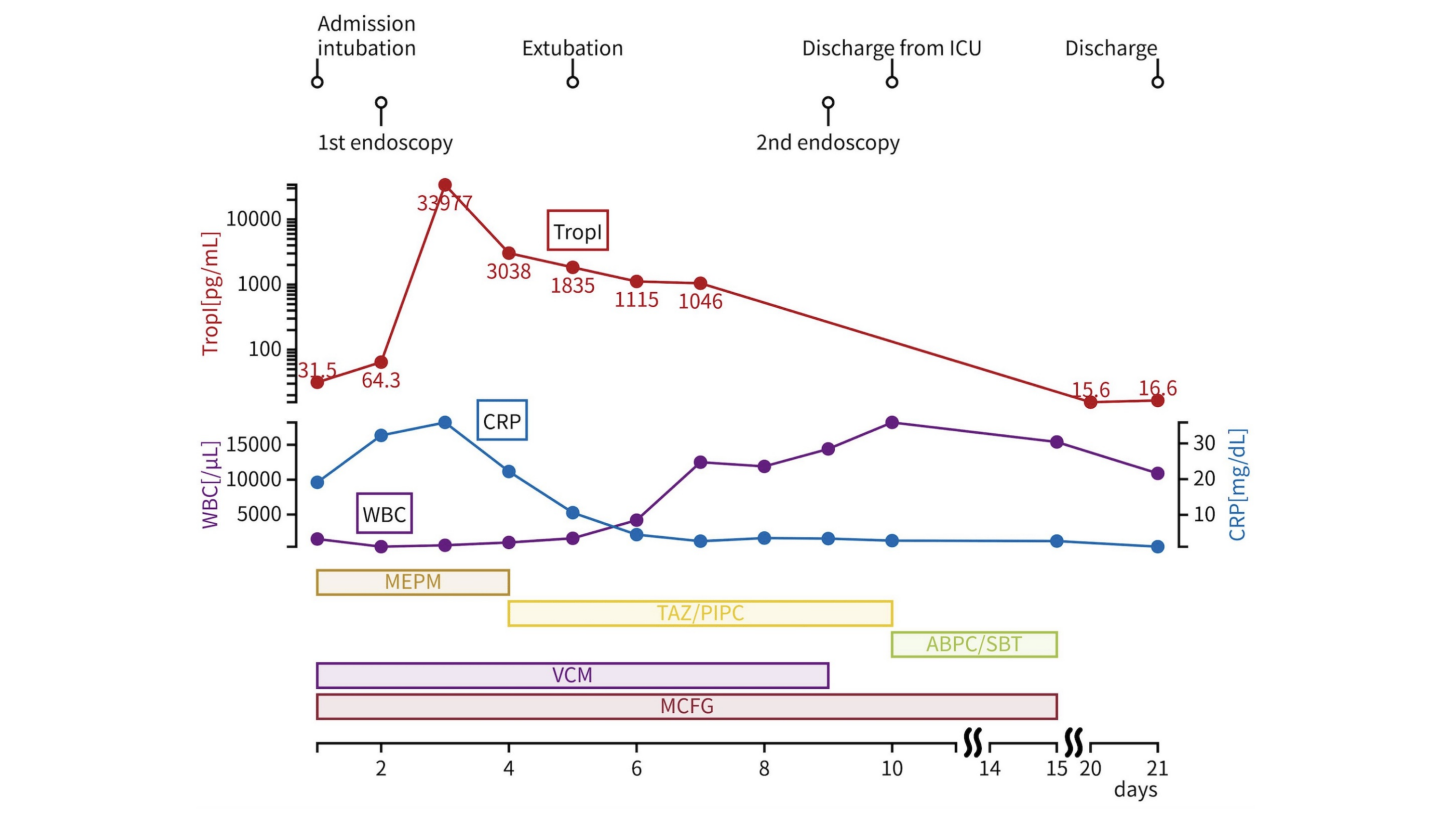
Temporal changes in troponin I, C-reactive protein (CRP), and white blood cell (WBC) counts with antibiotic administration timeline

On day 4 of hospitalization, the troponin I level increased to 33,977 pg/mL (reference value, <30 pg/mL). However, echocardiography revealed nothing of concern, and no further treatment was performed. The septic shock showed signs of improvement, allowing discontinuation of the continuous norepinephrine and vasopressin infusions, and the MEPM was de-escalated to tazobactam/piperacillin (TAZ/PIPC). Sedatives were tapered from day 5 of hospitalization; however, the patient remained unresponsive. Given the patient’s background of PBC and autoimmune hepatitis with elevated ammonia levels, we suspected hepatic encephalopathy, although the serum liver function tests were within normal ranges: aspartate aminotransferase (AST), 63 U/L; alanine aminotransferase (ALT), 92 U/L; and total-bilirubin (T-Bil), 1.25 mg/dL. Treatment with branched-chain amino acids and kanamycin improved patient responsiveness after 1 d, allowing for extubation. By day 7 of hospitalization, the troponin I level and ECG profile had normalized, and ECG and echocardiographic monitoring were discontinued. On day 8 of hospitalization, blood cultures were negative, and the VCM was discontinued. Figure 3 presents the temporal trends of serum troponin I, C-reactive protein (CRP), and white blood cell (WBC) counts, along with the timeline of administered antimicrobial agents. This figure underscores the relationship between inflammatory marker dynamics and therapeutic interventions, demonstrating a progressive resolution of systemic inflammation and myocardial involvement during the course of hospitalization.

On day 9 of hospitalization, an esophageal biopsy revealed numerous filamentous structures suggestive of *Candida* species. Species-level identification, such as via polymerase chain reaction (PCR), was not performed. However, based on the clinical presentation, endoscopic findings, histological evidence, and the patient’s favorable response to antifungal therapy, further testing was deemed unnecessary for guiding clinical management. Follow-up chest CT and upper gastrointestinal endoscopy revealed signs of improvement (Figures 1, 2B). On day 10 of hospitalization, oral intake was gradually initiated in small volumes, and TPN was discontinued. The TAZ/PIPC was de-escalated to ampicillin/sulbactam (AMPC/SBT). On day 15 of hospitalization, the MCFG and AMPC/SBT were discontinued, ending the antibiotic therapy. The patient’s general condition was stable, and he was discharged on day 21 of hospitalization. Outpatient follow-up was continued by the rheumatology and gastroenterology departments for the management of Sjögren's syndrome, overlap syndrome of PBC, and autoimmune hepatitis.

## Discussion

Acute phlegmonous esophagitis, a rare and life-threatening subtype of phlegmonous enteritis, is characterized by diffuse transmural inflammation involving the submucosal and muscular layers of the esophagus [1,2]. This condition is exceptionally uncommon and carries a high risk of mortality, typically associated with bacterial pathogens such as *Streptococcus*, *Staphylococcus*, *Escherichia coli*, *Haemophilus influenzae*, *Proteus* spp., and *Clostridium* spp. [2,3]. Established risk factors include immunosuppression, chronic alcohol use, uncontrolled diabetes mellitus, advanced age, malnutrition, low socioeconomic status, and underlying malignancy [1,4].

To the best of our knowledge, this represents the first documented case of acute phlegmonous esophagitis caused by *Candida* species. While superficial *Candida* esophagitis is a well-recognized entity, particularly in immunocompromised individuals [5], the involvement of deeper esophageal layers such as the submucosa and muscularis propria has not previously been reported [6]. This case suggests that under certain immunosuppressive conditions, especially the combination of prolonged corticosteroid therapy and Sjögren’s syndrome*, Candida* may act as an invasive pathogen, capable of eliciting a phlegmonous inflammatory response. Histopathological confirmation of *Candida* in the esophageal wall, together with the endoscopic appearance of circumferential white plaques, provided strong diagnostic evidence.

The clinical presentation of acute phlegmonous esophagitis is often nonspecific and variable, ranging from sore throat and chest pain to dysphagia and septic shock [3,4]. In our case, the patient initially experienced progressive dysphagia, which rapidly evolved into acute chest pain and hemodynamic instability, consistent with a fulminant disease course. Recent advances in imaging and endoscopic modalities have enabled earlier and more accurate diagnoses, which are critical for improving patient outcomes [1]. In particular, early endoscopic assessment not only facilitated prompt diagnosis in our case but also allowed for targeted biopsy and identification of the fungal pathogen, thereby guiding the initiation of appropriate antifungal therapy.

Although conservative management with broad-spectrum antibiotics is generally considered the standard of care for acute phlegmonous esophagitis, surgical interventions such as thoracotomy or endoscopic drainage may become necessary in cases refractory to medical treatment. However, thoracotomy has been associated with high morbidity and mortality, with a reported mortality rate of 24.2% (odds ratio: 19.53; 95% CI: 1.33-282; p = 0.03) according to a recent systematic review [1], and endoscopic drainage protocols have not yet been standardized [4]. In our case, combination therapy with antifungal and antibacterial agents led to clinical improvement without the need for surgical intervention, suggesting that timely and targeted medical therapy may be sufficient even in cases involving fungal pathogens.

An important and unusual complication observed in this patient was the extension of inflammation to the pericardium and myocardium, as evidenced by ST-segment elevation on electrocardiography and elevated serum troponin I levels. Although rare, cardiac involvement can result from direct inflammatory extension or a systemic inflammatory response, and may manifest as pericarditis, myocarditis, or even cardiogenic shock. These findings underscore the importance of continuous cardiac monitoring, including serial electrocardiograms, cardiac enzyme measurements, and echocardiography, in patients with severe esophageal infections accompanied by chest symptoms. Although cardiac MRI was not performed due to the patient’s unstable condition, the possibility of sepsis-related myocardial dysfunction could not be excluded, and this represents a diagnostic limitation of the case.

In this case, species-level identification and antifungal susceptibility testing were not performed; however, they were deemed unnecessary based on the clinical course and histological findings. Furthermore, histopathological confirmation from resected esophageal tissue was not available due to the avoidance of surgical intervention. Taken together, these factors represent limitations of this report. In addition, the absence of long-term clinical follow-up data after discharge limits our ability to assess the patient’s recovery trajectory and potential late complications, which remains a further limitation of this report.

In summary, this case broadens the spectrum of known etiologies of acute phlegmonous esophagitis by identifying *Candida* as a rare but plausible causative organism, particularly in immunocompromised hosts. It emphasizes the diagnostic value of early endoscopic examination, the importance of considering fungal pathogens in the differential diagnosis, and the need for vigilant cardiac monitoring in complicated cases. Further accumulation of case reports and systematic investigations may be essential to establish diagnostic criteria, refine treatment strategies, and develop risk stratification tools for this rare but potentially fatal condition. Furthermore, given the rarity of deep *Candida* invasion in the esophageal wall, these findings should be interpreted with caution, and broad generalization should be avoided until further cases are reported.

## Conclusions

This case illustrates that *Candida* species may rarely serve as causative pathogens in acute phlegmonous esophagitis, particularly under conditions of immunosuppression. The presence of circumferential white plaques on endoscopy, together with histopathological confirmation, underscores the critical importance of early endoscopic evaluation and the inclusion of fungal infections in the differential diagnosis. Moreover, the extension of inflammation to the pericardium and myocardium highlights the necessity for continuous cardiac monitoring, including serial electrocardiograms, cardiac enzyme measurements, and echocardiography, to promptly detect complications such as myocarditis and pericarditis. Further accumulation of case reports and future studies is warranted to refine antifungal treatment strategies and establish standardized monitoring protocols, ultimately aiming to improve outcomes in patients with this rare but potentially fatal condition.
